# 4-{2-[4-(Dimethyl­amino)­phen­yl]ethen­yl}-1-methyl­pyridinium 2-amino-3,5-dimethyl­benzene­sulfonate monohydrate

**DOI:** 10.1107/S160053681200089X

**Published:** 2012-01-18

**Authors:** Liang Li, Xutang Tao, Huai Yang, Zhou Yang

**Affiliations:** a Department of Materials Physics and Chemistry, School of Materials Science and Engineering, University of Science and Technology Beijing, Beijing 100083, People’s Republic of China; b State Key Laboratory of Crystal Materials, Shandong University, Jinan, 250100, People’s Republic of China

## Abstract

In the crystal structure of the title compound, C_16_H_19_N_2_^+^·C_8_H_10_NO_3_S^−^·H_2_O, the cations and anions are linked by O—H⋯O and N—H⋯O hydrogen bonds, forming alternating layers parallel to the *ac* plane. An intra­molecular N—H⋯O hydrogen bond occurs in the anion. The crystal structure is further stabilized by π–π inter­actions, with centroid–centroid distances of 3.7240 (9) and 3.6803 (8) Å.

## Related literature

For the synthesis, see: Okada *et al.* (1990 ▶). For background to non-linear optical materials, see: Gu *et al.* (2003 ▶); Dorrer (2006 ▶); Yang, Mutter *et al.* (2007 ▶); Ruiz *et al.* (2006 ▶). For the effects of different substituents of benzene sulfonate on its non-linear optical properties, see: Okada *et al.* (2003 ▶); Yang, Wörle *et al.* (2007 ▶); Ogawa *et al.* (2008 ▶), Yang *et al.* (2005 ▶), Yang, Jazbinsek *et al.*, (2007 ▶). For bond-length data, see: Allen *et al.* (1987 ▶).
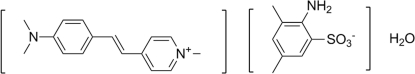


## Experimental

### 

#### Crystal data

C_16_H_19_N_2_^+^·C_8_H_10_NO_3_S^−^·H_2_O
*M* *_r_* = 457.58
Triclinic, 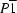

*a* = 8.7430 (17) Å
*b* = 9.5463 (19) Å
*c* = 14.109 (3) Å
α = 95.00 (3)°
β = 101.89 (3)°
γ = 96.29 (3)°
*V* = 1138.1 (4) Å^3^
*Z* = 2
Mo *K*α radiation
μ = 0.18 mm^−1^
*T* = 173 K
0.30 × 0.24 × 0.23 mm

#### Data collection

Rigaku MM007HF + CCD (Saturn724+) diffractometer
Absorption correction: multi-scan (*CrystalClear*; Rigaku, 2008 ▶) *T*_min_ = 0.713, *T*_max_ = 1.000
8126 measured reflections
3986 independent reflections
3722 reflections with *I* > 2σ(*I*)
*R*_int_ = 0.032

#### Refinement

*R*[*F* ^2^ > 2σ(*F* ^2^)] = 0.047
*wR*(*F* ^2^) = 0.122
*S* = 1.10
3986 reflections
294 parameters
H-atom parameters constrained
Δρ_max_ = 0.27 e Å^−3^
Δρ_min_ = −0.36 e Å^−3^

### 

Data collection: *CrystalClear* (Rigaku, 2008 ▶); cell refinement: *CrystalClear*; data reduction: *CrystalClear*; program(s) used to solve structure: *SHELXS97* (Sheldrick, 2008 ▶); program(s) used to refine structure: *SHELXL97* (Sheldrick, 2008 ▶); molecular graphics: *Mercury* (Macrae *et al.*, 2006 ▶); software used to prepare material for publication: *SHELXL97*.

## Supplementary Material

Crystal structure: contains datablock(s) I, global. DOI: 10.1107/S160053681200089X/zj2049sup1.cif

Supplementary material file. DOI: 10.1107/S160053681200089X/zj2049Isup2.cdx

Structure factors: contains datablock(s) I. DOI: 10.1107/S160053681200089X/zj2049Isup3.hkl

Supplementary material file. DOI: 10.1107/S160053681200089X/zj2049Isup4.cml

Additional supplementary materials:  crystallographic information; 3D view; checkCIF report

## Figures and Tables

**Table 1:** Hydrogen-bond geometry (Å, °)

*D*—H⋯*A*	*D*—H	H⋯*A*	*D*⋯*A*	*D*—H⋯*A*
O4—H4*B*⋯O3	0.84	2.01	2.854 (2)	179
O4—H4*A*⋯O2^i^	0.84	2.04	2.874 (2)	172
N3—H3*A*⋯O1	0.89	1.96	2.732 (2)	144
N3—H3*B*⋯O4^ii^	0.89	2.27	3.110 (3)	158

Symmetry codes: (i) 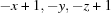; (ii) 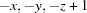.

**Fig. 1.**
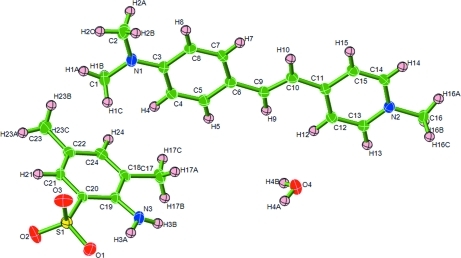
The molecular structure of the title compound showing 50% probability displacement ellipsoids.

**Fig. 2.**
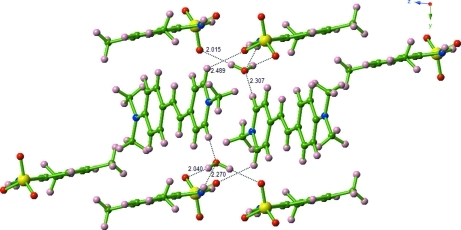
The crystal packing of the title organic salt, viewed down the y axis. Hydrogen bonds are shown as dashed lines.

## supplementary crystallographic information

### Comment

There has been intensive research on the development of nonlinear optical materials for its potential application in high-speed and high-density data processing, storage and telecommunications. (Gu *et al.*, 2003; Dorrer, 2006; Yang, Mutter *et al.*, 2007). The title compound was synthesized as part of our continuing research on the nonlinear optical properties of DAS (4-*N*,*N*-dimethylamino-4'-*N*'-methyl-stilbazolium) derivatives. (Okada *et al.*, 2003; Ogawa *et al.*, 2008; Yang *et al.*, 2005; Ruiz *et al.*, 2006; Yang Wörle *et al.*, 2007; Yang, Jazbinsek *et al.*, 2007). Fig. 1 illustrates the molecular structure of organic salt with the atomic numbering scheme. The bond distances and angles in both the cation and anion are in normal ranges (Allen *et al.*, 1987). The unit cell of the title compound contains two asymmetric units, each consisting of one C_16_H_19_N_2_^+^ cation, one C_8_H_5_O_7_S^-^ anion and one water molecule. In the crystal structure, atoms H3A and H3B are involved in N—H—O interactions, while atoms H4A and H4B are involved O—H—O hydrogen bonds. The distance of H3A—O1 and H3B—O4 are 1.96 Å and 2.27 Å, while the distance of H4B—O2 and H4B—O3 are 2.01 Å and 2.04 Å. The cations and anions are stacked in a parallel manner and form alternating layers. The crystal structure is further stabilized by π–π interactions with a *Cg*1···*Cg*1 and a *Cg*1···*Cg*2 separation of 3.6803 (8) Å and 3.7240 (9) Å, respectively (*Cg*1 and *Cg*2 are the centroids of the N2/C11—C15 pyridine ring and C3—C8 benzene ring, respectively).

### Experimental

4-*N*,*N*-dimethylamino-4'-*N*'-methyl-stilbazolium 2-amino-3,5-dimethylbenzenesulfonate was prepared by the metathesization of 4-*N*,*N*-dimethylamino-4'-*N*'-methyl-stilbazolium iodide (Okada *et al.*, 1990) with the sodium salt of the 2-amino-3,5-dimethylbenzenesulfonic acid. The title salt was then recrystallized from methanol to get high purity material for crystal growth. 4-{2-[4-(Dimethylamino)phenyl]ethenyl}-1-methylpyridinium 2-amino-3,5-dimethylbenzenesulfonate monohydrate: yield 65%; ^1^H-NMR (400 MHz, DMSO-d6): delta=8.68 (d, 2H, J= 6.8 Hz, C_5_H_4_N), 8.04(d, 2H, J= 6.8 Hz, C_5_H_4_N), 7.92 (d, 1H, J=16.0 Hz, CH), 7.60(d, 2H, J= 8.4 Hz, C_6_H_4_), 7.18 (m, 2H, J= 16.0 Hz, CH+C_6_H_2_SO_3_^-^), 6.80 (d, 2H, J= 8.8 Hz, C_6_H_4_), 6.73(s, 1H, C_6_H_4_SO_3_^-^),5.18(s, 2H, NH_2_), 4.16(s, 3H, NMe), 3.01 (s, 6H, NMe_2_), 2.10(s, 3H, Me), 2.01(s, 3H, Me). C, H, N analysis calcd. for C_24_H_24_N_2_O_7_S: C 65.58, H 6.65, N 9.56; found: C 65.60,H 6.67, N 9.53. Crystals were obtained by slow cooling method from 45^O^C to room temperature in methanol: first the saturated solution of the title compound in methanol at 45°C was prepared. Spontaneous nucleation could be observed after cooling down the saturated solution. Then the temperature was increased to dissolve most of the nuclei and made sure that only one or two nucleated crystals remained undissolved. After that, large crystals with good quality for X-Ray measurements could be obtained by slow cooling the solution at the temperature of about 34°C.

### Refinement

All H atoms were located geometrically (methyl C—H = 0.98 Å, aromatic C—H = 0.95 Å, N—H =0.89 Å and O—H = 0.84 Å) and refined using a riding model, with *U*_iso_(H) = 1.2 or 1.5*U*_eq_(C).

### Crystal data

**Table:**

C_16_H_19_N_2_^+^·C_8_H_10_NO_3_S^−^·H_2_O	*Z* = 2
*M**_r_* = 457.58	*F*(000) = 488
Triclinic, *P*1	*D*_x_ = 1.335 Mg m^−^^3^
Hall symbol: -p 1	Mo *K*α radiation, λ = 0.71073 Å
*a* = 8.7430 (17) Å	Cell parameters from 4072 reflections
*b* = 9.5463 (19) Å	θ = 1.5–27.5°
*c* = 14.109 (3) Å	µ = 0.18 mm^−^^1^
α = 95.00 (3)°	*T* = 173 K
β = 101.89 (3)°	Block, red
γ = 96.29 (3)°	0.30 × 0.24 × 0.23 mm
*V* = 1138.1 (4) Å^3^	

### Data collection

**Table:**

Rigaku MM007HF + CCD (Saturn724+) diffractometer	3986 independent reflections
Radiation source: Rotating Anode	3722 reflections with *I* > 2σ(*I*)
Confocal	*R*_int_ = 0.032
Detector resolution: 28.5714 pixels mm^-1^	θ_max_ = 25.0°, θ_min_ = 2.8°
ω scans at fixed χ = 45°	*h* = −10→10
Absorption correction: multi-scan (*CrystalClear*; Rigaku, 2008)	*k* = −11→10
*T*_min_ = 0.713, *T*_max_ = 1.000	*l* = −13→16
8126 measured reflections	

### Refinement

**Table:**

Refinement on *F*^2^	Primary atom site location: structure-invariant direct methods
Least-squares matrix: full	Secondary atom site location: difference Fourier map
*R*[*F*^2^ > 2σ(*F*^2^)] = 0.047	Hydrogen site location: inferred from neighbouring sites
*wR*(*F*^2^) = 0.122	H-atom parameters constrained
*S* = 1.10	*w* = 1/[σ^2^(*F*_o_^2^) + (0.0533*P*)^2^ + 0.5973*P*] where *P* = (*F*_o_^2^ + 2*F*_c_^2^)/3
3986 reflections	(Δ/σ)_max_ < 0.001
294 parameters	Δρ_max_ = 0.27 e Å^−^^3^
0 restraints	Δρ_min_ = −0.36 e Å^−^^3^

### Special details

**Table:**

Geometry. All e.s.d.'s (except the e.s.d. in the dihedral angle between two l.s. planes) are estimated using the full covariance matrix. The cell e.s.d.'s are taken into account individually in the estimation of e.s.d.'s in distances, angles and torsion angles; correlations between e.s.d.'s in cell parameters are only used when they are defined by crystal symmetry. An approximate (isotropic) treatment of cell e.s.d.'s is used for estimating e.s.d.'s involving l.s. planes.
Refinement. Refinement of *F*^2^ against ALL reflections. The weighted *R*-factor *wR* and goodness of fit *S* are based on *F*^2^, conventional *R*-factors *R* are based on *F*, with *F* set to zero for negative *F*^2^. The threshold expression of *F*^2^ > σ(*F*^2^) is used only for calculating *R*-factors(gt) *etc*. and is not relevant to the choice of reflections for refinement. *R*-factors based on *F*^2^ are statistically about twice as large as those based on *F*, and *R*- factors based on ALL data will be even larger.

### Fractional atomic coordinates and isotropic or equivalent isotropic displacement parameters (Å^2^)

**Table:**

	*x*	*y*	*z*	*U*_iso_*/*U*_eq_	
S1	0.35960 (6)	−0.01854 (5)	0.64442 (4)	0.02452 (16)	
O1	0.25159 (18)	−0.09428 (18)	0.55886 (11)	0.0414 (4)	
O2	0.47733 (18)	−0.10464 (18)	0.68633 (12)	0.0413 (4)	
O3	0.4275 (2)	0.12009 (16)	0.62811 (12)	0.0415 (4)	
O4	0.33542 (17)	0.21476 (16)	0.44082 (11)	0.0359 (4)	
H4B	0.3642	0.1870	0.4958	0.043*	
H4A	0.3960	0.1802	0.4087	0.043*	
N1	−0.1077 (2)	0.43155 (18)	0.10981 (14)	0.0323 (4)	
N2	1.14453 (19)	0.57343 (18)	0.40983 (12)	0.0250 (4)	
N3	−0.0089 (2)	−0.02843 (19)	0.62299 (13)	0.0313 (4)	
H3A	0.0435	−0.0563	0.5784	0.038*	
H3B	−0.1114	−0.0597	0.6115	0.038*	
C1	−0.1983 (3)	0.5453 (2)	0.08007 (17)	0.0340 (5)	
H1C	−0.1826	0.6198	0.1348	0.051*	
H1A	−0.3105	0.5078	0.0601	0.051*	
H1B	−0.1630	0.5849	0.0253	0.051*	
C2	−0.1884 (3)	0.2877 (2)	0.0917 (2)	0.0419 (6)	
H2A	−0.1506	0.2345	0.0405	0.063*	
H2C	−0.3022	0.2896	0.0708	0.063*	
H2B	−0.1670	0.2417	0.1517	0.063*	
C3	0.0515 (2)	0.4578 (2)	0.14488 (14)	0.0246 (4)	
C4	0.1328 (2)	0.5964 (2)	0.15746 (15)	0.0272 (4)	
H4	0.0761	0.6733	0.1407	0.033*	
C5	0.2934 (2)	0.6218 (2)	0.19382 (15)	0.0284 (5)	
H5	0.3447	0.7163	0.2012	0.034*	
C6	0.3837 (2)	0.5134 (2)	0.22030 (14)	0.0249 (4)	
C7	0.3029 (2)	0.3751 (2)	0.20748 (14)	0.0261 (4)	
H7	0.3600	0.2986	0.2245	0.031*	
C8	0.1428 (2)	0.3481 (2)	0.17085 (15)	0.0265 (4)	
H8	0.0922	0.2532	0.1628	0.032*	
C9	0.5511 (2)	0.5491 (2)	0.26169 (14)	0.0264 (4)	
H9	0.5922	0.6465	0.2676	0.032*	
C10	0.6549 (2)	0.4597 (2)	0.29268 (15)	0.0267 (4)	
H10	0.6164	0.3616	0.2870	0.032*	
C11	0.8219 (2)	0.5027 (2)	0.33428 (14)	0.0249 (4)	
C12	0.8933 (2)	0.6450 (2)	0.35356 (15)	0.0276 (4)	
H12	0.8308	0.7194	0.3410	0.033*	
C13	1.0521 (2)	0.6765 (2)	0.39030 (14)	0.0266 (4)	
H13	1.0984	0.7729	0.4024	0.032*	
C14	1.0799 (2)	0.4359 (2)	0.39385 (14)	0.0268 (4)	
H14	1.1451	0.3637	0.4080	0.032*	
C15	0.9215 (2)	0.3997 (2)	0.35749 (15)	0.0269 (4)	
H15	0.8782	0.3025	0.3478	0.032*	
C16	1.3155 (2)	0.6116 (2)	0.45006 (16)	0.0323 (5)	
H16C	1.3328	0.6552	0.5175	0.049*	
H16B	1.3593	0.6789	0.4109	0.049*	
H16A	1.3678	0.5260	0.4484	0.049*	
C17	−0.1655 (2)	0.0252 (2)	0.77634 (18)	0.0343 (5)	
H17A	−0.2024	0.0886	0.7280	0.051*	
H17C	−0.1968	0.0533	0.8373	0.051*	
H17B	−0.2123	−0.0725	0.7518	0.051*	
C18	0.0114 (2)	0.0350 (2)	0.79461 (16)	0.0264 (4)	
C19	0.0835 (2)	0.00344 (19)	0.71531 (14)	0.0237 (4)	
C20	0.2495 (2)	0.01369 (19)	0.73519 (14)	0.0228 (4)	
C21	0.3380 (2)	0.0561 (2)	0.82936 (15)	0.0257 (4)	
H21	0.4496	0.0628	0.8407	0.031*	
C22	0.2692 (2)	0.0888 (2)	0.90665 (15)	0.0280 (5)	
C23	0.3656 (3)	0.1396 (3)	1.00757 (17)	0.0408 (6)	
H23A	0.4755	0.1241	1.0108	0.061*	
H23C	0.3235	0.0868	1.0551	0.061*	
H23B	0.3607	0.2410	1.0225	0.061*	
C24	0.1045 (3)	0.0758 (2)	0.88675 (16)	0.0288 (5)	
H24	0.0541	0.0959	0.9391	0.035*	

### Atomic displacement parameters (Å^2^)

**Table:**

	*U*^11^	*U*^22^	*U*^33^	*U*^12^	*U*^13^	*U*^23^
S1	0.0209 (3)	0.0246 (3)	0.0281 (3)	0.00316 (19)	0.0056 (2)	0.0024 (2)
O1	0.0290 (8)	0.0526 (10)	0.0371 (9)	0.0016 (7)	0.0053 (7)	−0.0147 (8)
O2	0.0332 (9)	0.0554 (10)	0.0448 (10)	0.0240 (8)	0.0159 (7)	0.0172 (8)
O3	0.0585 (11)	0.0287 (8)	0.0407 (9)	−0.0035 (7)	0.0239 (8)	0.0034 (7)
O4	0.0296 (8)	0.0451 (9)	0.0367 (9)	0.0135 (7)	0.0090 (7)	0.0098 (7)
N1	0.0238 (9)	0.0268 (9)	0.0430 (11)	0.0035 (7)	−0.0011 (8)	0.0057 (8)
N2	0.0225 (9)	0.0290 (9)	0.0237 (9)	0.0037 (7)	0.0060 (7)	0.0018 (7)
N3	0.0206 (9)	0.0381 (10)	0.0331 (10)	0.0039 (7)	0.0018 (7)	0.0027 (8)
C1	0.0278 (11)	0.0359 (12)	0.0390 (13)	0.0078 (9)	0.0047 (10)	0.0100 (10)
C2	0.0283 (12)	0.0344 (13)	0.0567 (16)	−0.0016 (10)	−0.0028 (11)	0.0071 (11)
C3	0.0235 (10)	0.0276 (10)	0.0230 (10)	0.0034 (8)	0.0054 (8)	0.0045 (8)
C4	0.0268 (11)	0.0246 (10)	0.0316 (11)	0.0062 (8)	0.0071 (9)	0.0050 (8)
C5	0.0289 (11)	0.0231 (10)	0.0329 (11)	0.0017 (8)	0.0070 (9)	0.0029 (8)
C6	0.0241 (10)	0.0294 (11)	0.0224 (10)	0.0043 (8)	0.0080 (8)	0.0011 (8)
C7	0.0275 (11)	0.0258 (10)	0.0272 (10)	0.0085 (8)	0.0074 (8)	0.0050 (8)
C8	0.0282 (11)	0.0230 (10)	0.0281 (11)	0.0013 (8)	0.0066 (9)	0.0024 (8)
C9	0.0270 (11)	0.0249 (10)	0.0279 (11)	0.0024 (8)	0.0086 (9)	0.0019 (8)
C10	0.0259 (10)	0.0247 (10)	0.0303 (11)	0.0021 (8)	0.0085 (9)	0.0024 (8)
C11	0.0265 (10)	0.0275 (10)	0.0227 (10)	0.0048 (8)	0.0087 (8)	0.0045 (8)
C12	0.0286 (11)	0.0272 (11)	0.0278 (11)	0.0076 (8)	0.0057 (9)	0.0046 (8)
C13	0.0284 (11)	0.0242 (10)	0.0269 (10)	0.0032 (8)	0.0061 (8)	0.0013 (8)
C14	0.0290 (11)	0.0263 (10)	0.0270 (10)	0.0086 (8)	0.0078 (9)	0.0033 (8)
C15	0.0280 (11)	0.0233 (10)	0.0306 (11)	0.0031 (8)	0.0087 (9)	0.0047 (8)
C16	0.0222 (11)	0.0418 (13)	0.0310 (11)	0.0036 (9)	0.0029 (9)	0.0005 (9)
C17	0.0238 (11)	0.0316 (12)	0.0502 (14)	0.0053 (9)	0.0119 (10)	0.0081 (10)
C18	0.0232 (10)	0.0196 (10)	0.0382 (12)	0.0044 (8)	0.0089 (9)	0.0059 (8)
C19	0.0228 (10)	0.0179 (9)	0.0307 (11)	0.0033 (8)	0.0049 (8)	0.0058 (8)
C20	0.0214 (10)	0.0179 (9)	0.0290 (10)	0.0037 (7)	0.0042 (8)	0.0044 (8)
C21	0.0204 (10)	0.0245 (10)	0.0313 (11)	0.0033 (8)	0.0027 (8)	0.0042 (8)
C22	0.0315 (11)	0.0239 (10)	0.0283 (11)	0.0062 (8)	0.0042 (9)	0.0039 (8)
C23	0.0431 (14)	0.0459 (14)	0.0325 (12)	0.0128 (11)	0.0040 (10)	0.0002 (10)
C24	0.0339 (11)	0.0236 (10)	0.0335 (11)	0.0076 (9)	0.0152 (9)	0.0051 (9)

### Geometric parameters (Å, °)

**Table:**

S1—O3	1.4470 (16)	C9—C10	1.347 (3)
S1—O1	1.4492 (17)	C9—H9	0.9500
S1—O2	1.4507 (16)	C10—C11	1.455 (3)
S1—C20	1.779 (2)	C10—H10	0.9500
O4—H4B	0.8400	C11—C15	1.399 (3)
O4—H4A	0.8401	C11—C12	1.411 (3)
N1—C3	1.365 (3)	C12—C13	1.368 (3)
N1—C2	1.450 (3)	C12—H12	0.9500
N1—C1	1.454 (3)	C13—H13	0.9500
N2—C13	1.352 (3)	C14—C15	1.368 (3)
N2—C14	1.352 (3)	C14—H14	0.9500
N2—C16	1.478 (3)	C15—H15	0.9500
N3—C19	1.373 (3)	C16—H16C	0.9800
N3—H3A	0.8902	C16—H16B	0.9800
N3—H3B	0.8900	C16—H16A	0.9800
C1—H1C	0.9800	C17—C18	1.505 (3)
C1—H1A	0.9800	C17—H17A	0.9800
C1—H1B	0.9800	C17—H17C	0.9800
C2—H2A	0.9800	C17—H17B	0.9800
C2—H2C	0.9800	C18—C24	1.382 (3)
C2—H2B	0.9800	C18—C19	1.419 (3)
C3—C4	1.410 (3)	C19—C20	1.411 (3)
C3—C8	1.413 (3)	C20—C21	1.394 (3)
C4—C5	1.379 (3)	C21—C22	1.379 (3)
C4—H4	0.9500	C21—H21	0.9500
C5—C6	1.399 (3)	C22—C24	1.399 (3)
C5—H5	0.9500	C22—C23	1.506 (3)
C6—C7	1.405 (3)	C23—H23A	0.9800
C6—C9	1.451 (3)	C23—H23C	0.9800
C7—C8	1.376 (3)	C23—H23B	0.9800
C7—H7	0.9500	C24—H24	0.9500
C8—H8	0.9500		
			
O3—S1—O1	113.26 (11)	C15—C11—C12	116.18 (19)
O3—S1—O2	112.91 (11)	C15—C11—C10	119.85 (18)
O1—S1—O2	111.58 (10)	C12—C11—C10	123.98 (18)
O3—S1—C20	105.38 (9)	C13—C12—C11	120.40 (19)
O1—S1—C20	107.64 (9)	C13—C12—H12	119.8
O2—S1—C20	105.41 (9)	C11—C12—H12	119.8
H4B—O4—H4A	102.7	N2—C13—C12	121.43 (19)
C3—N1—C2	120.82 (18)	N2—C13—H13	119.3
C3—N1—C1	120.90 (17)	C12—C13—H13	119.3
C2—N1—C1	117.98 (17)	N2—C14—C15	120.57 (19)
C13—N2—C14	119.89 (17)	N2—C14—H14	119.7
C13—N2—C16	119.89 (17)	C15—C14—H14	119.7
C14—N2—C16	120.22 (17)	C14—C15—C11	121.51 (19)
C19—N3—H3A	113.8	C14—C15—H15	119.2
C19—N3—H3B	123.0	C11—C15—H15	119.2
H3A—N3—H3B	117.1	N2—C16—H16C	109.5
N1—C1—H1C	109.5	N2—C16—H16B	109.5
N1—C1—H1A	109.5	H16C—C16—H16B	109.5
H1C—C1—H1A	109.5	N2—C16—H16A	109.5
N1—C1—H1B	109.5	H16C—C16—H16A	109.5
H1C—C1—H1B	109.5	H16B—C16—H16A	109.5
H1A—C1—H1B	109.5	C18—C17—H17A	109.5
N1—C2—H2A	109.5	C18—C17—H17C	109.5
N1—C2—H2C	109.5	H17A—C17—H17C	109.5
H2A—C2—H2C	109.5	C18—C17—H17B	109.5
N1—C2—H2B	109.5	H17A—C17—H17B	109.5
H2A—C2—H2B	109.5	H17C—C17—H17B	109.5
H2C—C2—H2B	109.5	C24—C18—C19	119.59 (18)
N1—C3—C4	121.40 (18)	C24—C18—C17	121.22 (19)
N1—C3—C8	121.85 (18)	C19—C18—C17	119.19 (19)
C4—C3—C8	116.75 (18)	N3—C19—C20	123.04 (18)
C5—C4—C3	120.87 (19)	N3—C19—C18	119.28 (18)
C5—C4—H4	119.6	C20—C19—C18	117.60 (18)
C3—C4—H4	119.6	C21—C20—C19	120.54 (18)
C4—C5—C6	122.42 (19)	C21—C20—S1	115.75 (15)
C4—C5—H5	118.8	C19—C20—S1	123.67 (15)
C6—C5—H5	118.8	C22—C21—C20	122.29 (19)
C5—C6—C7	116.73 (18)	C22—C21—H21	118.9
C5—C6—C9	119.24 (18)	C20—C21—H21	118.9
C7—C6—C9	124.00 (19)	C21—C22—C24	116.84 (19)
C8—C7—C6	121.48 (19)	C21—C22—C23	122.0 (2)
C8—C7—H7	119.3	C24—C22—C23	121.1 (2)
C6—C7—H7	119.3	C22—C23—H23A	109.5
C7—C8—C3	121.73 (18)	C22—C23—H23C	109.5
C7—C8—H8	119.1	H23A—C23—H23C	109.5
C3—C8—H8	119.1	C22—C23—H23B	109.5
C10—C9—C6	127.36 (19)	H23A—C23—H23B	109.5
C10—C9—H9	116.3	H23C—C23—H23B	109.5
C6—C9—H9	116.3	C18—C24—C22	123.12 (19)
C9—C10—C11	124.62 (19)	C18—C24—H24	118.4
C9—C10—H10	117.7	C22—C24—H24	118.4
C11—C10—H10	117.7		
			
C2—N1—C3—C4	176.3 (2)	N2—C14—C15—C11	1.0 (3)
C1—N1—C3—C4	2.7 (3)	C12—C11—C15—C14	−2.0 (3)
C2—N1—C3—C8	−4.1 (3)	C10—C11—C15—C14	177.92 (19)
C1—N1—C3—C8	−177.65 (19)	C24—C18—C19—N3	−175.99 (18)
N1—C3—C4—C5	179.38 (19)	C17—C18—C19—N3	3.2 (3)
C8—C3—C4—C5	−0.3 (3)	C24—C18—C19—C20	0.8 (3)
C3—C4—C5—C6	−0.3 (3)	C17—C18—C19—C20	−179.99 (17)
C4—C5—C6—C7	0.5 (3)	N3—C19—C20—C21	175.45 (18)
C4—C5—C6—C9	−177.54 (19)	C18—C19—C20—C21	−1.2 (3)
C5—C6—C7—C8	−0.1 (3)	N3—C19—C20—S1	−2.0 (3)
C9—C6—C7—C8	177.76 (19)	C18—C19—C20—S1	−178.72 (14)
C6—C7—C8—C3	−0.4 (3)	O3—S1—C20—C21	−72.98 (17)
N1—C3—C8—C7	−179.06 (19)	O1—S1—C20—C21	165.88 (15)
C4—C3—C8—C7	0.6 (3)	O2—S1—C20—C21	46.66 (17)
C5—C6—C9—C10	178.8 (2)	O3—S1—C20—C19	104.61 (18)
C7—C6—C9—C10	0.9 (3)	O1—S1—C20—C19	−16.53 (19)
C6—C9—C10—C11	−179.54 (19)	O2—S1—C20—C19	−135.75 (17)
C9—C10—C11—C15	−175.28 (19)	C19—C20—C21—C22	0.4 (3)
C9—C10—C11—C12	4.7 (3)	S1—C20—C21—C22	178.10 (15)
C15—C11—C12—C13	1.8 (3)	C20—C21—C22—C24	0.8 (3)
C10—C11—C12—C13	−178.17 (19)	C20—C21—C22—C23	−177.79 (19)
C14—N2—C13—C12	−0.6 (3)	C19—C18—C24—C22	0.4 (3)
C16—N2—C13—C12	−179.62 (18)	C17—C18—C24—C22	−178.75 (18)
C11—C12—C13—N2	−0.5 (3)	C21—C22—C24—C18	−1.2 (3)
C13—N2—C14—C15	0.4 (3)	C23—C22—C24—C18	177.38 (19)
C16—N2—C14—C15	179.38 (18)		

### Hydrogen-bond geometry (Å, °)

**Table:**

*D*—H···*A*	*D*—H	H···*A*	*D*···*A*	*D*—H···*A*
O4—H4B···O3	0.84	2.01	2.854 (2)	179
O4—H4A···O2^i^	0.84	2.04	2.874 (2)	172
N3—H3A···O1	0.89	1.96	2.732 (2)	144
N3—H3B···O4^ii^	0.89	2.27	3.110 (3)	158

Symmetry codes: (i) −*x*+1, −*y*, −*z*+1; (ii) −*x*, −*y*, −*z*+1.
